# 621 Ethical Considerations to Prevent Burns in Patients Who Smoke While Receiving Home Oxygen Therapy

**DOI:** 10.1093/jbcr/irac012.249

**Published:** 2022-03-23

**Authors:** Kimberly H Khoo, Joshua S Yoon, Charles S Hultman, Julie Caffrey

**Affiliations:** Department of Plastic and Reconstructive Surgery, Johns Hopkins University School of Medicine, Baltimore, Maryland; R. Adams Cowley Shock Trauma Center, Division of Plastic Reconstructive and Maxillofacial Surgery, Herndon, Virginia; Johns Hopkins University School of Medicine, Baltimore, Maryland; Johns Hopkins, Baltimore, Maryland

## Abstract

**Introduction:**

Home oxygen therapy (HOT) is prescribed to patients with pulmonary dysfunction as a means of improving survival and quality of life. However, ignition of HOT can lead to burns that carry significant morbidity and mortality. This is especially true for patients who actively smoke while on HOT, with prior studies showing that 87.3% of HOT-related burns were due to smoking. An ethical issue thus arises for providers who routinely treat this patient population: how to balance providing beneficial treatment for a patient with the responsibility to protect that patient from suffering unnecessary burn injuries.

**Methods:**

A literature review was conducted to determine the conversations surrounding this ethical dilemma in the scientific community. Thought was given to various solutions to address issues, and each solution was analyzed with respect to the traditional ethical principles of autonomy, beneficence, nonmaleficence, and justice.

**Results:**

Multiple prevention considerations should be made for this patient population of active smokers on HOT. The first is to encourage more judicious prescription of home oxygen. This approach supports the principle of nonmaleficence as utilizing more prudence with prescription may prevent unnecessarily putting a patient at risk. A second strategy would be to discontinue a patient’s HOT if they were found to be an active smoker despite proper education; however, this solution conflicts with beneficence, especially if the patient relies heavily on HOT. A third, more drastic approach would be to withhold treatment of patients who repeatedly present with burns acquired secondary to smoking while on HOT. Refusal to treat conflicts not only with beneficence and maleficence but also with justice, as social factors play into which patients are more likely to smoke and, thus, may present with smoking-related burns while on HOT. Lastly, it is possible to address this challenge with broader, upstream solutions such as more thorough, longitudinal patient education on smoking cessation and the risks of smoking while on HOT. By doing so, physicians support all four of the traditional ethical principles.

**Conclusions:**

Patients who suffer from burn injuries secondary to HOT ignition present a unique ethical challenge. Though physicians are tasked with the duty to provide optimal care for these patients, they are also shouldered with the responsibility of patient education and advocacy. Physicians should address this outstanding dilemma by thinking more critically about potential solutions that are bolstered by ethical considerations.

